# Sex differences in the association of overweight/obesity with asthma and lung function in adolescents aged 10–18 years: a meta-analysis

**DOI:** 10.3389/fped.2026.1889125

**Published:** 2026-10-27

**Authors:** Siyu Wang, Yu Zhang, Xiaoming Huang, Yao Tian

**Affiliations:** 1 Department of General Pediatrics, West China Second University Hospital, Sichuan University, Chengdu, Sichuan, China; 2 Key Laboratory of Birth Defects and Related Diseases of Women and Children (Sichuan University), Ministry of Education, Chengdu, Sichuan, China

**Keywords:** adolescent, asthma, meta-analysis, obesity, overweight, pulmonary function

## Abstract

**Objective:**

The global prevalence of overweight, obesity, and asthma in adolescents has increased rapidly. However, previous studies have reported inconsistent correlations between adiposity and respiratory indicators, and only a few meta-analyses have focused on sex-based disparities. In this study, we systematically assessed the associations of overweight and obesity with asthma and pulmonary function among adolescents aged 10–18 years and explored sex differences.

**Methods:**

This study followed Preferred Reporting Items for Systematic Reviews and Meta-Analyses 2020 (PRISMA 2020) guidelines. Eligible observational studies were retrieved and screened. Odds ratios (ORs) were pooled for asthma, and standardized mean differences (SMDs; Hedges’ *g*) were pooled for pulmonary function outcomes using random-effects models. Sex differences were evaluated with formal interaction tests. Subgroup, leave-one-out sensitivity, and Egger's regression analyses were performed, and evidence certainty was rated using Grading of Recommendations Assessment, Development, and Evaluation (GRADE).

**Results:**

Twelve studies were pooled for asthma: overweight and obesity were associated with higher odds of asthma incidence (OR = 1.26, 95% CI: 1.15–1.37), with significant regional (*P* for interaction < 0.001) and sex (*P* = 0.010) interactions; the association was stronger in females (OR = 2.11, 95% CI: 1.44–3.10) than in males (OR = 1.07, 95% CI: 0.75–1.52). For pulmonary function (six studies), the overweight/obesity group showed higher forced vital capacity (FVC; SMD = 0.35, 95% CI: 0.09–0.61) and forced expiratory volume in 1 s (FEV1; SMD = 0.23, 95% CI: 0.01–0.45) but lower FEV1/FVC (SMD = −0.27, 95% CI: −0.36 to −0.18), with no significant sex interaction for any outcome. No significant differences were observed for peak expiratory flow (PEF), forced expiratory flow at 75% of FVC (FEF75), or forced expiratory flow between 25% and 75% of FVC (FEF25–75). Small-study effects in the asthma pool were largely attributable to the regional subgroup structure. Four outcomes were rated moderate certainty and seven low certainty by GRADE.

**Conclusion:**

Overweight and obesity are associated with higher odds of asthma incidence and an early obstructive pulmonary function pattern in adolescents. The asthma association is stronger in females, whereas no sex differences were detected for pulmonary function. Targeted screening of high-risk adolescent girls and further large-scale multicenter studies are warranted.

**Systematic Review Registration:**

https://www.crd.york.ac.uk/PROSPERO/view/CRD420261398331, PROSPERO CRD420261398331.

## 1 Introduction

In recent years, overweight and obesity among children and adolescents have become increasingly prevalent globally, emerging as a major public health concern. Driven by shifting lifestyles, widespread consumption of high-calorie and high-fat diets, increased sedentary behavior, and insufficient physical activity, the prevalence of overweight and obesity in children and adolescents has been increasing globally over the past two decades, featuring a younger onset age and worldwide spread (1–4). Meanwhile, bronchial asthma ranks among the most prevalent chronic non-communicable respiratory diseases in adolescents, and its global incidence continues to climb every year. Recurrent wheezing, cough, and airway hyperresponsiveness severely impair motor ability, physical development, and mental health in adolescents and also increase the global medical burden, drawing extensive attention from the international public health community (5, 6). Adolescence is a critical period of rapid development of respiratory structure, pulmonary ventilation function, and systemic metabolism. Airway sensitivity, immune inflammatory status, and body fat distribution present distinct age-specific characteristics in adolescents, which differ substantially from those in adults (7). Free from confounding factors such as long-term smoking, occupational exposure, and chronic underlying diseases that are common in middle-aged and elderly populations, adolescents serve as an ideal research population to objectively reflect the actual correlation of overweight and obesity with asthma onset and pulmonary function changes (8).

Existing epidemiological and clinical studies have verified the close correlation of overweight and obesity with asthma incidence and pulmonary function impairment in adolescents (9–11). Excessive fat accumulation in the thoracic and abdominal cavities pushes up the diaphragm and reduces thoracic compliance, thereby directly reducing the lung volume and ventilation capacity (12). In addition, obesity-induced chronic low-grade systemic inflammation and dysregulated adipokines trigger and aggravate airway inflammation and hyperresponsiveness, further facilitating asthma onset, progression, and poor disease control (13, 14).

Numerous studies have explored the association of overweight and obesity with asthma and pulmonary function in adolescents, yet evident limitations and controversies remain. Discrepancies in racial backgrounds, geographical regions, age ranges, sample sizes, and asthma diagnostic criteria lead to inconsistent research conclusions (15). Sex differences in sex hormone levels, fat distribution patterns, and sensitivity to inflammatory response during puberty result in heterogeneous impacts of obesity on asthma and pulmonary function (16, 17). Most existing meta-analyses fail to conduct a quantitative pooled analysis stratified by sex, leaving the correlation characteristics across sex unclear (18, 19).

Pulmonary function indicators such as forced vital capacity (FVC), forced expiratory volume in 1 s (FEV1), the FEV1/FVC ratio, and peak expiratory flow (PEF) are continuous variables. Original studies commonly apply linear regression and report the regression coefficient β to quantify the magnitude of their associations with adiposity. Traditional meta-analyses primarily adopt odds ratios (ORs) only for dichotomous disease endpoints, which cannot accurately reflect the linear dose–response relationship between body mass index (BMI) changes and continuous pulmonary function parameters. Simple forced conversion of effect sizes may result in information loss and increase pooled estimation bias (20). Moreover, the regression coefficients reported by original studies are derived from incomparable exposure units, outcome scales, and covariate adjustment sets, making direct pooling of β across studies methodologically inappropriate. Standardized mean differences (SMDs) calculated from group-level data provide a comparable effect-size metric for continuous outcomes measured on different scales. Accordingly, this meta-analysis pooled odds ratios for asthma and SMD (Hedges’ *g*) for pulmonary function outcomes.

Based on the above contradictory findings and methodological shortcomings of previous meta-analyses, we put forward the following study hypothesis: overweight and obesity are associated with asthma risk and altered pulmonary function in adolescents, and such associations differ significantly between male and female adolescents.

The primary aim of this meta-analysis was to quantitatively synthesize the overall association between overweight, obesity, and asthma incidence among adolescents aged 10–18 years.

The secondary objectives were as follows: (1) to compare sex-specific correlations between adiposity and asthma risk and (2) to evaluate linear relationships of overweight and obesity with multiple continuous pulmonary function markers, including FVC, FEV1, FEV1/FVC, and PEF-related small-airway indicators via SMD (Hedges’ *g*) calculated from group-level data.

In this review, we conducted only sex stratification rather than subgroup analysis by pubertal stage or obesity grade. Sex difference is the most neglected research gap supported by consistent biological evidence (16, 17), whereas most of the included studies lack unified Tanner staging and BMI grading data for reliable pooling. Most of the included studies recorded complete sex information to provide more comprehensive data for analysis, making sex our primary stratification factor.

Given the above research gaps, the pooled estimates generated in this work deliver multilayered practical benefits for diverse stakeholders. For clinicians, sex-specific quantitative associations can guide targeted screening and early weight intervention for adolescent patients with asthma. For public health policymakers, synthesized evidence supports the formulation of population-level prevention strategies against co-occurring obesity and asthma among youths. For school health managers and other relevant practitioners, our findings provide evidence-based references for global adolescent obesity prevention, early asthma screening, and individualized respiratory health management.

## 2 Materials and methods

### 2.1 Literature search

This study was conducted in strict accordance with the Preferred Reporting Items for Systematic Reviews and Meta-Analyses 2020 (PRISMA 2020) guidelines, covering literature retrieval, screening, quality evaluation, and meta-analysis. This meta-analysis was prospectively registered with the International Prospective Register of Systematic Reviews (PROSPERO; registration no.: CRD420261398331). Relevant studies concerning the effects of overweight and obesity on adolescent asthma were retrieved from the China National Knowledge Infrastructure (CNKI), Wanfang, VIP, Chinese Biomedical Literature Database (CBM), PubMed, Cochrane Library, Web of Science, and Embase by combining subject terms and free words (detailed retrieval strategies in Supplementary Material 1). The search time ranged from the establishment of database establishment to 1 May 2026. ClinicalTrials.gov and the International Clinical Trials Registry Platform (ICTRP; initiated by the World Health Organization [WHO]) were also searched to collect information on ongoing clinical trials.

### 2.2 Inclusion and exclusion criteria

#### 2.2.1 Inclusion criteria

1. Study types included case–control studies, cohort studies, and cross-sectional studies.
2. Subjects were adolescents aged 10–18 years, with or without asthma.
3. Studies contained groups with normal weight, overweight, and obesity.
4. Reported asthma and/or pulmonary function outcomes.
5. The literature was published in Chinese or English.

#### 2.2.2 Exclusion criteria

1. Patients complicated with other respiratory diseases or severe systemic underlying diseases.
2. Studies lacking extractable data for the outcomes of interest (asthma and/or pulmonary function).
3. Duplicate publications and overlapping retrieved literature.
4. Literature with incomplete data unavailable for valid analysis.
5. Papers without accessible full texts or retracted publications.
6. Case reports, reviews, conference abstracts, and animal experimental studies.

### 2.3 Literature screening and data extraction

Two independent reviewers performed literature screening and data extraction in duplicate. All retrieved records from various databases were imported into EndNote X9 (Clarivate, Philadelphia, PA, USA) for deduplication. Titles and abstracts were preliminarily screened, and eligible full texts were subsequently obtained. Qualified studies in the literature that met the inclusion and exclusion criteria were selected for data extraction. Any disagreement was resolved through mutual discussion or adjudication by a third reviewer. Extracted data mainly covered first author, publication year, country, sample size, asthma prevalence, and pulmonary function indicators.

### 2.4 Literature quality assessment

Two reviewers independently assessed the risk of bias in the included studies. The Newcastle–Ottawa Scale (NOS) (21) was adopted to evaluate the quality of case–control and cohort studies.

The scale assesses eight dimensions: representativeness of the exposed cohort, selection of the non-exposed cohort, ascertainment of exposure, absence of baseline outcome differences, intergroup comparability, outcome assessment, follow-up duration, and follow-up completeness. The total score ranges from 0 to 9. Studies scoring 0–3 points are defined as low quality, 4–6 points as moderate quality, and 7–9 points as high quality. In the studies, discrepancies were settled by a discussion or judgment from a third reviewer.

### 2.5 Statistical analysis

Statistical analyses were performed using STATA software, version 14.0 (StataCorp LLC, College Station, TX, USA). Odds ratio was pooled for dichotomous outcomes (22, 23). For continuous pulmonary function outcomes, group-level means, SDs, 95% confidence interval (CI), and sample sizes were extracted for adiposity-exposed and comparison groups. When overweight and obesity were reported separately, the groups were combined using standard formulas for pooled means and standard deviations. When medians and interquartile ranges were reported, means and standard deviations were approximated using the method proposed by Wan and colleagues (24). SMDs were calculated as Hedges’ *g* with small sample correction, with positive values indicating higher pulmonary function values in the overweight/obesity group than in the normal weight group (25, 26). Random-effects models were used because clinical and methodological heterogeneity was anticipated across adiposity definitions, populations, pulmonary function scales, and study designs (27). One independent effect was used from each study in the overall analyses. For studies reporting only sex-stratified group data, the effects of sex differences were first combined within the study using fixed-effects inverse-variance weighting to avoid double counting; sex-stratified estimates were then analyzed separately (28). Heterogeneity was quantified using Cochran's *Q*, the corresponding *P*-value, and *I*^2^ (29). Formal sex interaction tests were performed by dividing the difference between male and female random-effects pooled estimates by the standard error of that difference (30). Statistical significance was defined as a two-sided *P*-value below 0.05. A leave-one-out sensitivity analysis was performed to verify the stability of the pooled results. Publication bias was assessed by a visual inspection of funnel plots and Egger's regression test. For outcomes with at least 10 studies, funnel plot asymmetry was further corrected using the trim-and-fill method. For outcomes with fewer than 10 studies, Egger's test was performed exploratorily and interpreted with caution because of its limited statistical power. The quality of pooled evidence was graded using the Grading of Recommendations Assessment, Development and Evaluation (GRADE).

## 3 Results

### 3.1 Literature screening results

A total of 2,189 articles were initially retrieved, and 18 studies involving 211,452 participants were ultimately included (Figure 1) (31–48).

**Figure 1:**
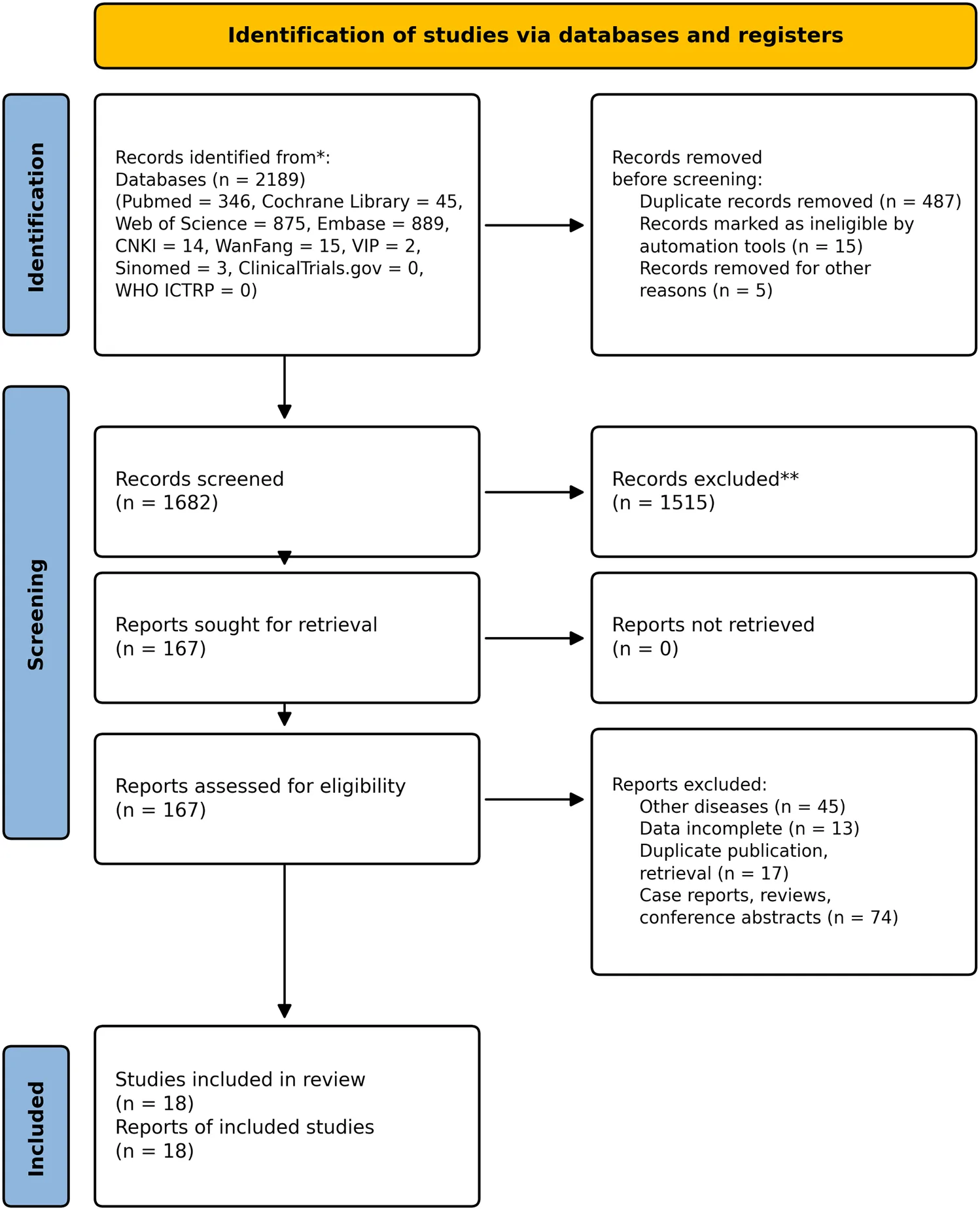
A PRISMA 2020 flow diagram of the literature screening and study selection process.

The 18 included studies comprised two case–control studies and 16 cohort studies (Table 1). Three studies were carried out in Mainland China, five in Taiwan region, three in the United States, two in Sweden, and two in Italy. The rest were from the United Kingdom, South Africa, and the Netherlands. Nine outcome indicators were analyzed in total.

**Table 1:** Characteristics of the 18 studies included in the meta-analysis of adolescents aged 10–18 years.

Study	Type of study	Country	Sample size		Age		Sex (male)		Outcome indicators	Quality evaluation
			Case group	Control group	Case group	Control group	Case group	Control group		
Lingyu et al. (31)	Cohort study	China	507	511	10.3 ± 2.4	10.3 ± 2.4	260	262	a	9
Jing (32)	Cohort study	China	2,048	5,274	12.30 ± 2.14	1,283	3,765	a,b,c,d,e,i	7	
Zhaoxia et al. (33)	Case–control study	China	155	953	10.6 ± 0.7	10.8 ± 0.6	129	620	a,b,c,d,e,f,g,h,i	9
Aris et al. (34)	Cohort study	America	–	–	13.2 ± 0.9	–	a,b,c,d	6		
Chen et al. (35)	Cohort study	Taiwan	1,908	5,284	14.48 ± 0.57	936	2,733	b,c,d	8	
Chen et al. (36)	Cohort study	Taiwan	2,649	5,274	12.5 ± 0.4	1,076	–	a	8	
Chu et al. (37)	Cohort study	Taiwan	1,829	12,825	14.21 ± 0.92	14.30 ± 0.92	969	6,257	a	8
Cibella et al. (38)	Cohort study	Italy	933	1,460	12.52 ± 1.07	12.41 ± 0.91	509	424	b,c,d	8
Ekström (39)	Cohort study	Sweden	3,115	–	12–14	12–14	–	d	7	
Engwa et al. (40)	Cohort study	South Africa	77	463	10–14	10–14	33	207	a,b,c,d,e,i	8
Ho et al. (41)	Cohort study	Taiwan	1,013	3,039	13–15	13–15	572	1,634	a	7
Kalm et al. (42)	Cohort study	Sweden	84	711	13.5 ± 0.42	13.5 ± 0.40	49	345	a	8
Kang and Sui (43)	Cohort study	Taiwan	150	795	14.35 ± 0.20	14.54 ± 0.10	71	398	a	7
Lang et al. (44)	Cohort study	America	40,900	1,16,469	12–17	12–17	19,951	52,839	a	8
Rhee et al. (45)	Cohort study	America	146	148	–	86	67	a,b,c	6	
Sposato et al. (46)	Case–control study	Italy	130	669	16.6 ± 1.3	16.5 ± 1.4	96	438	a,b,c,d	7
Wang et al. (47)	Cohort study	England	149	497	11.34 ± 0.53	79	260	a,b,c	9	
Wu et al. (48)	Cohort study	Netherlands	1,493	1,384	10–13	10–13	–	a,b,c,d,h	8	

a, Asthma; b, FVC; c, FEV1; d, FEV1/FVC; e, PEF; f, FEF25; g, FEF50; h, FEF75; i, FEF25−75.

### 3.2 Quality assessment results

Quality assessment using the NOS rated 16 studies as high quality (three studies scoring 9, eight studies scoring 8, and five studies scoring 7 points) and the remaining two studies as moderate quality (6 points) (Table 1; Supplementary Material 1). No low-quality literature was identified.

Most included studies possessed sound methodological quality with a low risk of bias, which was suitable for a pooled effect size analysis. Three studies failed to obtain scores in the representativeness assessment of the exposed cohort because of an unclear description of population source representativeness. Overall, the enrolled studies had reliable quality, limited bias, and credible research findings.

### 3.3 Meta-analysis results of the association of overweight and obesity with asthma

Fifteen studies exploring the association between overweight, obesity, and asthma were included. Among them, 12 studies (31–34, 36, 37, 40, 43, 44, 46–48) with complete effect size data were pooled for analysis (Table 2; forest plots in Supplementary Material 2). A random-effects model was used as prespecified; heterogeneity was found to be high (*I*^2^ = 84%, *P* < 0.001). The pooled result indicated that overweight and obesity were associated with increased odds of asthma incidence (OR = 1.26, 95% CI: 1.15–1.37, *P* < 0.001).

**Table 2:** Meta-analysis of overweight/obesity with asthma and pulmonary function, overall and stratified by sex, with GRADE ratings.

Outcome indicators	Number of studies	Heterogeneity test		Model of effect	SMD (95% CI)	Overall *P*-value	*P* for sex interaction	GRADE
		*P*	*I*^2^ (%)					
Asthma	12	<0.001	84	Random	1.26 (1.15, 1.37)^a^	<0.001	–	Low
Asthma—Taiwan	3	0.274	23	Random	1.04 (1.01, 1.06)^a^	0.002	<0.001^b^	Moderate
Asthma—other	9	0.112	39	Random	1.44 (1.29, 1.62)^a^	<0.001		Moderate
Asthma—male	2	0.675	0	Random	1.07 (0.75, 1.52)^a^	0.718	0.01	Low
Asthma—female	3	0.323	11	Random	2.11 (1.44, 3.10)^a^	<0.001		Moderate
FVC	6	<0.001	98	Random	0.35 (0.09, 0.61)	0.008	0.66	Low
FVC—male	3	<0.001	98	Random	0.25 (−0.15, 0.65)	0.23		
FVC—female	3	<0.001	98	Random	0.38 (−0.02, 0.77)	0.06		
FEV1	6	<0.001	97	Random	0.23 (0.01, 0.45)	0.04	0.42	Low
FEV1—male	3	<0.001	97	Random	0.11 (−0.17, 0.40)	0.43		
FEV1—female	3	<0.001	97	Random	0.29 (−0.02, 0.60)	0.07		
FEV1/FVC	6	<0.001	81	Random	−0.27 (−0.36, −0.18)	<0.001	0.47	Moderate
FEV1/FVC—male	3	<0.001	96	Random	−0.31 (−0.56, −0.06)	0.01		Low
FEV1/FVC—female	3	<0.001	92	Random	−0.20 (−0.38, −0.01)	0.04		
PEF	3	0.004	82	Random	0.02 (−0.18, 0.22)	0.85	0.75	Low
PEF—male	1	–	–	–	−0.05 (−0.12, 0.02)	0.13		
PEF—female	1	–	–	–	−0.07 (−0.15, 0.01)	0.09		
FEF25−75	4	0.015	71	Random	−0.01 (−0.12, 0.09)	0.79	0.68	Low
FEF25−75—male	2	0.223	33	Random	−0.08 (−0.15, 0.00)	0.05		
FEF25−75—female	2	0.035	77	Random	−0.04 (−0.19, 0.11)	0.59		
FEF75	1	–	–	–	0.02 (−0.15, 0.19)	0.84	–	Low

a OR (95% CI).

b *P* for region interaction.

#### 3.3.1 Subgroup analysis

Subgroup analyses stratified by study region and by sex were performed to explore the sources of heterogeneity, and subgroup differences were assessed using formal interaction tests.

##### 3.3.1.1 Region subgroup

Studies were divided into a Taiwan subgroup [three studies (36, 37, 43)] and other-regions subgroup [nine studies (31–34, 40, 44, 46–48)]. In the Taiwan subgroup, heterogeneity was low (*I*^2^ = 23%, *P* = 0.27), and overweight/obesity showed a weak association with asthma (OR = 1.04, 95% CI: 1.01–1.06, *P* = 0.002). In the other-regions subgroup, heterogeneity was moderate (*I*^2^ = 39%, *P* = 0.11), and overweight/obesity was associated with markedly increased odds of asthma (OR = 1.44, 95% CI: 1.29–1.62, *P* < 0.001; Figure 2). The association differed significantly between the two regional subgroups (*P* for interaction < 0.001), indicating that region was a major source of the observed heterogeneity.

**Figure 2:**
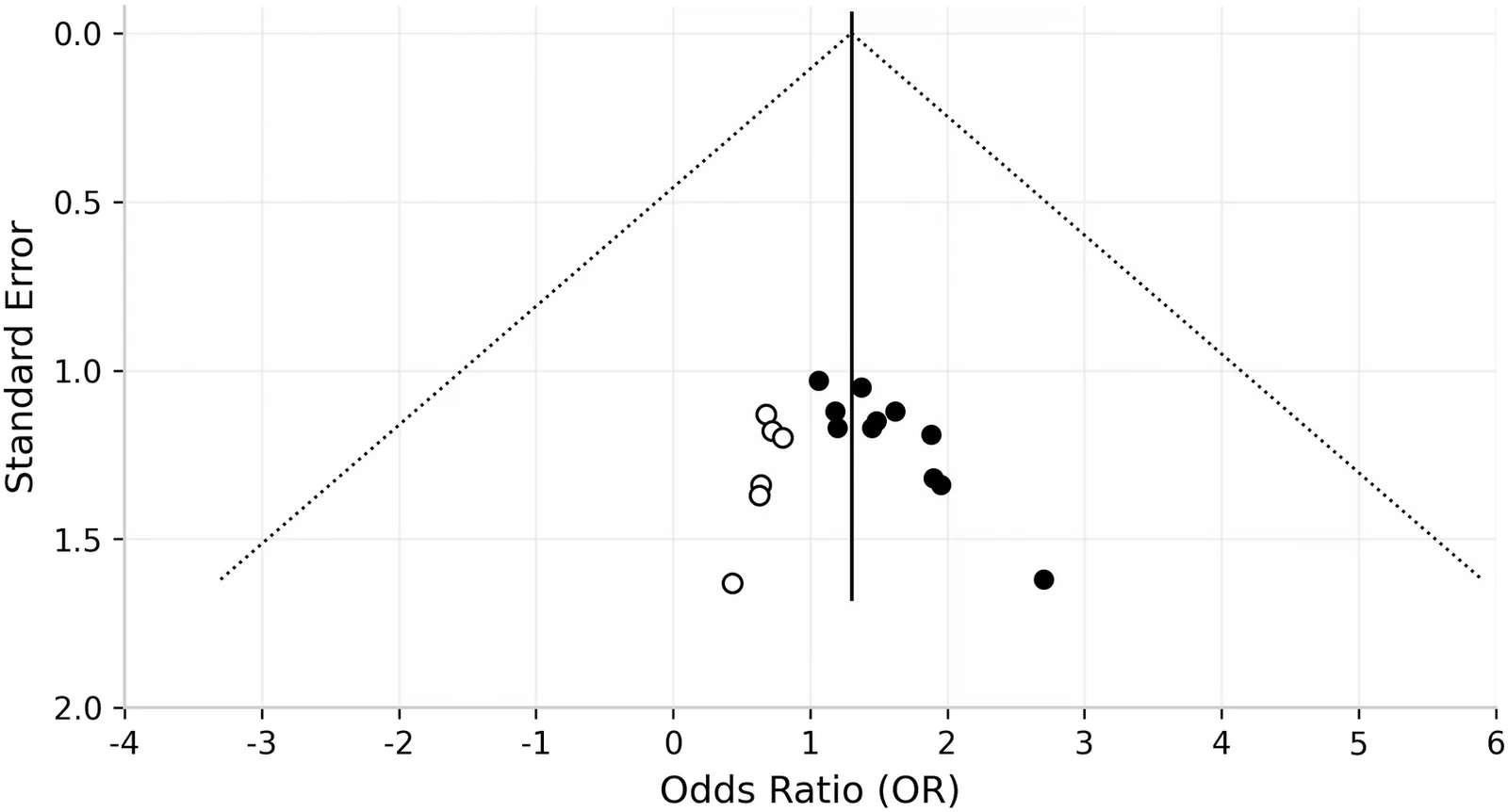
The association of overweight and obesity with asthma in adolescents: overall and stratified by geographic region (random-effects OR, 95% CI).

##### 3.3.1.2 Sex subgroup

Two studies (41, 47) were included for male adolescents, with no observed heterogeneity (*I*^2^ = 0%, *P* = 0.675); the pooled association was not statistically significant (OR = 1.07, 95% CI: 0.75–1.52, *P* = 0.718). Three studies (41, 42, 47) were included for female adolescents and had low heterogeneity (*I*^2^ = 11%, *P* = 0.323); overweight and obesity were associated with significantly increased odds of asthma incidence in females (OR = 2.11, 95% CI: 1.44–3.10, *P* < 0.001; Figure 3). The association was significantly stronger in females than in males (ratio of ORs = 1.98, 95% CI: 1.18–3.33; *P* for interaction = 0.010), and this was confirmed in a within-study contrast restricted to the two studies reporting both sexes (ratio of ORs = 1.89, 95% CI: 1.13–3.14; *P* = 0.014) (41, 47).

**Figure 3:**
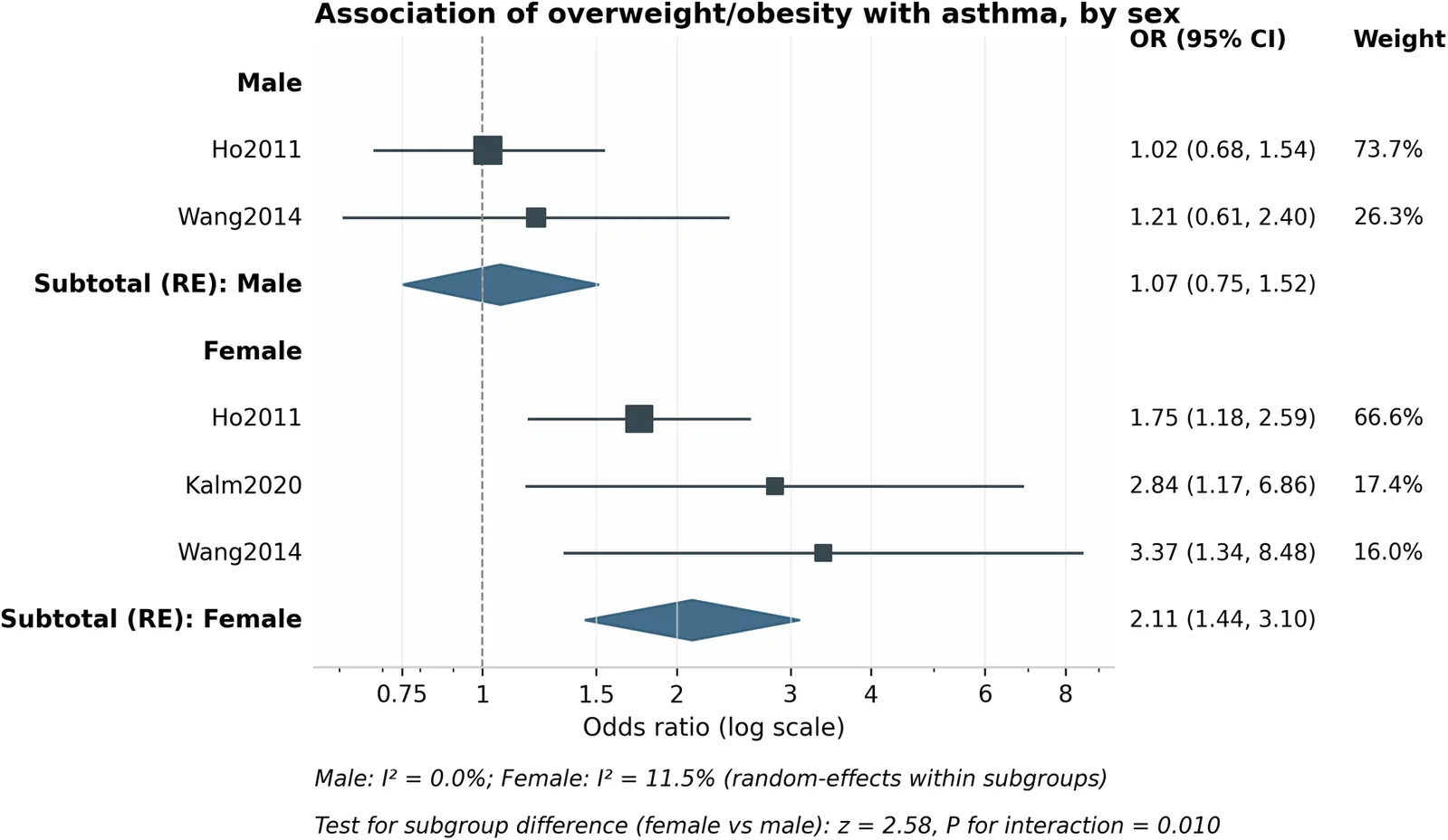
Sex-stratified association between overweight/obesity and asthma in adolescents aged 10–18 years (random-effects OR, 95% CI).

### 3.4 Meta-analysis of overweight, obesity, and pulmonary function in adolescents

Because pulmonary function outcomes were reported on incomparable scales and units across studies, SMDs (Hedges’ *g*) comparing the overweight/obesity group with the normal weight or non-obesity group were calculated and pooled using random-effects models, rather than pooling regression coefficients. Six studies (32, 33, 35, 38, 40, 46) provided eligible group-level data for the volumetric outcomes. The study populations included general schoolchildren, children with suspected asthma or airway hyperresponsiveness, and adolescents with asthma.

#### 3.4.1 Forced vital capacity

Six studies (32, 33, 35, 38, 40, 46) were pooled, and heterogeneity was found to be very high (*I*^2^ = 98%, *P* < 0.001). FVC was significantly higher in the overweight/obesity group than in the comparison group (SMD = 0.35, 95% CI: 0.09–0.61, *P* = 0.008), and the direction of effect was positive in five of the six studies.

#### 3.4.2 Forced expiratory volume in 1 s

Six studies (32, 33, 35, 38, 40, 46) were pooled (*I*^2^ = 97%, *P* < 0.001). FEV1 was higher in the overweight/obesity group with a borderline significant pooled estimate (SMD = 0.23, 95% CI: 0.01–0.45, *P* = 0.040).

#### 3.4.3 FEV1/FVC

Six studies (32, 33, 35, 38, 40, 46) were pooled (*I*^2^ = 81%, *P* < 0.001). FEV1/FVC was significantly lower in the overweight/obesity group (SMD = −0.27, 95% CI: −0.36 to −0.18, *P* < 0.001), and the direction of effect was consistent across all six studies.

#### 3.4.4 Peak expiratory flow and related indicators

##### 3.4.4.1 Peak expiratory flow

Three studies (32, 33, 40) were pooled (*I*^2^ = 82%, *P* = 0.004). No significant difference in PEF was found between the overweight/obesity and the comparison groups (SMD = 0.02, 95% CI: −0.18 to 0.22, *P* = 0.851).

Forced expiratory flow at 75% of FVC (FEF75): Only one study (33) reported FEF75, with no significant between-group difference (SMD = 0.02, 95% CI: −0.15 to 0.19, *P* = 0.841); this result is only descriptive.

Forced expiratory flow between 25% and 75% of FVC (FEF25–75): Four studies (32, 33, 38, 40) were pooled (*I*^2^ = 71%, *P* = 0.015). No significant difference was observed (SMD = −0.01, 95% CI: −0.12 to 0.09, *P* = 0.790). Given the small number of studies, these results should be interpreted cautiously.

### 3.5 Meta-analysis results of pulmonary function in overweight and obese adolescents stratified by sex

#### 3.5.1 Forced vital capacity

Three studies (32, 35, 38) provided sex-stratified data. In males, the pooled SMD was 0.25 (95% CI: −0.15 to 0.65, *P* = 0.227; *I*^2^ = 99%), while in females, it was 0.38 (95% CI: −0.02 to 0.77, *P* = 0.063; *I*^2^ = 98%). No significant sex interaction was detected (*P* for interaction = 0.658).

#### 3.5.2 Forced expiratory volume in 1 s

Three studies (32, 35, 38) were included. The pooled SMD was 0.11 (95% CI: −0.17 to 0.40, *P* = 0.434) in males and 0.29 (95% CI: −0.02 to 0.60, *P* = 0.069) in females, with no significant sex interaction (*P* for interaction = 0.419).

#### 3.5.3 FEV1/FVC

Three studies (32, 35, 38) were included. In the overweight/obesity group, FEV1/FVC was significantly lower in both males (SMD = −0.31, 95% CI: −0.56 to −0.06, *P* = 0.014) and females (SMD = −0.20, 95% CI: −0.38 to −0.01, *P* = 0.037), with no significant sex interaction (*P* for interaction = 0.468).

#### 3.5.4 PEF and FEF25–75

Only one study (32) reported sex-stratified PEF (male SMD = −0.05, 95% CI: −0.12 to 0.02; female SMD = −0.07, 95% CI: −0.15 to 0.01; within-study sex contrast *P* = 0.754). Two studies (32, 38) reported sex-stratified FEF25–75, with pooled SMDs of −0.08 (95% CI: −0.15 to 0.00, *P* = 0.051) in males and −0.04 (95% CI: −0.19 to 0.11, *P* = 0.587) in females and no significant sex interaction (*P* for interaction = 0.678).

In summary, no statistically significant sex interaction was detected for any pulmonary function outcome (all *P* for interaction > 0.05), although these tests had limited statistical power given the small number of studies contributing sex-stratified data.

### 3.6 Sensitivity analysis

Leave-one-out sensitivity analyses were performed for all pooled outcomes (Supplementary Material 3). The study by Sun et al. (32) was the major source of heterogeneity for the volumetric outcomes: after its removal, heterogeneity decreased substantially for FVC (*I*^2^ from 98% to 70%; SMD = 0.44, 95% CI: 0.34–0.54, *P* < 0.001), FEV1 (*I*^2^ from 97% to 55%; SMD = 0.31, 95% CI: 0.23–0.39, *P* < 0.001), and FEV1/FVC (*I*^2^ from 81% to 25%; SMD = −0.32, 95% CI: −0.38 to −0.27, *P* < 0.001), while all three pooled estimates remained statistically significant and directionally unchanged. The pooled FEV1 estimate lost statistical significance when Chen (35), Cibella (38), Engwa (40), or Wu (33) was individually omitted and should therefore be interpreted with caution. The FEV1/FVC result was robust in all leave-one-out iterations.

### 3.7 Publication bias

Funnel plot asymmetry was assessed for the asthma outcome. Egger's test indicated significant small-study effects for the overall asthma pool (*P* < 0.001), which were largely attributable to the regional subgroup structure (*P* = 0.074 within the other-regions subgroup). The trim-and-fill method was used to adjust the pooled estimate; after supplementing six hypothetical studies, we found that the adjusted OR was 1.095 (95% CI: 1.001–1.197), suggesting that the association remained directionally stable (Figure 4). For pulmonary function outcomes, Egger's tests were not significant (FVC *P* = 0.647, FEV1 *P* = 0.597, FEV1/FVC *P* = 0.946, and FEF25–75 *P* = 0.148), although power was limited given the small number of studies.

**Figure 4:**
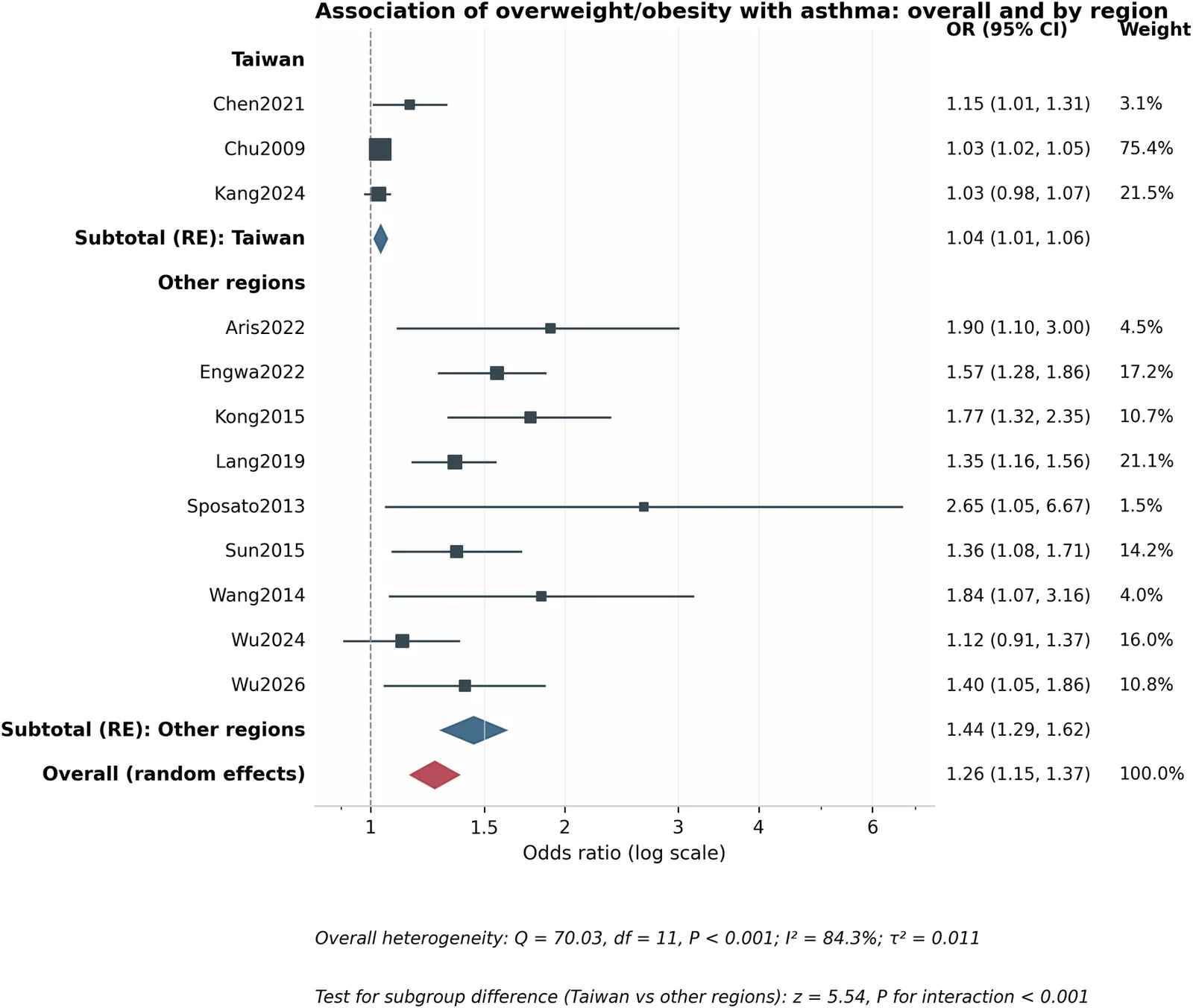
A trim-and-fill funnel plot assessing small-study effects in the asthma meta-analysis.

### 3.8 GRADE evidence quality assessment

The GRADE system was adopted to evaluate the evidence quality of all pooled outcome indicators (49). Following GRADE guidance for non-randomized studies, the body of evidence started at a level of high certainty, was rated down for risk of bias, inconsistency, indirectness, imprecision, and publication bias, and was rated up for large effects (50). Four outcomes were rated as moderate certainty (asthma in the Taiwan and other-regions subgroups, asthma in females, and FEV1/FVC), the last of these supported by a consistent direction of effect across all six studies and a precise pooled estimate. Seven outcomes were rated as low certainty (asthma overall, asthma in males, FVC, FEV1, PEF, FEF25–75, and FEF75), mainly because of substantial unexplained heterogeneity, indirectness from mixed asthma endpoint definitions and populations, imprecision from a few contributing studies, or suspected small-study effects (Table 2, Supplementary Material 1). Sex-stratified pulmonary function estimates were rated as low certainty because of imprecision from the small number of contributing studies.

## 4 Discussion

This meta-analysis systematically explored the associations of overweight and obesity with asthma onset and pulmonary function indicators among adolescents aged 10–18 years and further clarified sex-stratified intergroup differences. Our pooled results tentatively suggest that overweight and obesity are associated with elevated asthma risk in adolescents, a trend partially consistent with previous population-based studies (18, 19, 51–53). From a shared pathophysiological perspective, excess visceral and thoracic fat may elevate the diaphragm and impair thoracic compliance, thereby altering ventilatory mechanics. Meanwhile, adipose tissue continuously secretes multiple inflammatory mediators to trigger chronic low-grade systemic inflammation, which may chronically irritate the airways, augment airway hyperresponsiveness, and increase asthma susceptibility (54–56). This pathological pathway is further supported by previous basic and clinical observations (57, 58).

A sex-stratified analysis revealed a statistically significant sex interaction for asthma (*P* for interaction = 0.010): the association was evident in female adolescents (OR = 2.11, 95% CI: 1.44–3.10) but not in male adolescents (OR = 1.07, 95% CI: 0.75–1.52). This finding differs from that in the meta-analysis by Ahmadizar et al. (19), which focused on childhood obesity, poor asthma control, and exacerbations and did not identify an evident sex-modifying effect; this discrepancy may arise from differences in participant age ranges and the asthma endpoint definitions adopted by each study. Physiologically, adolescent females are more prone to excessive accumulation of chest wall and visceral fat (59). Excessive fat deposition restricts thoracic mobility and reduces the pulmonary dilatation reserve (60). In addition, multiple inflammatory factors secreted by the adipose tissue induce systemic low-grade inflammation. Combined with the regulatory effect of estrogen on airway immunity during puberty, airway hyperresponsiveness is aggravated, thereby increasing susceptibility to asthma (61). In contrast, male adolescents tend to have more skeletal muscle and a lower body fat proportion, and androgens may produce anti-inflammatory and bronchodilatory actions that partially counteract obesity-related respiratory damage (62, 63). Nevertheless, this sex-stratified finding should be interpreted as exploratory: only two studies contributed data on male adolescents, the asthma endpoints differed across the contributing studies (incident asthma, incident wheeze, and asthma risk), and the GRADE certainty was moderate for the female subgroup but low for the male subgroup. Further large-scale studies with sex-stratified reporting are required for validation.

The core pulmonary function indexes included FVC, FEV1, FEV1/FVC, PEF, FEF75, and FEF25–75 (64). FVC represents maximal ventilatory reserve and thoracic expansion capacity; FEV1 evaluates large airway ventilatory efficiency and expiratory muscle strength. FEV1/FVC acts as the core indicator for screening obstructive ventilatory dysfunction. PEF, FEF75, and FEF25–75 reflect the overall expiratory output, upper airway status, and mid-to-small airway function, respectively, which are widely adopted to assess small airway damage and airway hyperresponsiveness among adolescents (65, 66).

Pooled SMD showed that overweight and obesity were associated with higher FVC and FEV1 and lower FEV1/FVC, consistent with the findings reported by Winck et al. (67). Elevated FVC and FEV1 may reflect adaptive pulmonary compensation induced by fat-mediated thoracic restriction (59, 68). Excess visceral and thoracic fat pushes the diaphragm upward and limits thoracic expansion, and it has been hypothesized that the lung compensatorily increases the vital capacity to sustain baseline ventilation, thereby generating higher FVC and FEV1 readings (60). Meanwhile, chronic low-grade inflammation caused by obesity may infiltrate the airway mucosa, may trigger smooth muscle hyperplasia and mucosal edema, and may accelerate premature airway collapse during expiration. The declined expiratory flow forms an early subclinical obstructive ventilatory feature, corresponding to decreased FEV1/FVC (61). Notably, the direction of effect on FEV1/FVC was consistent across all six included studies and remained robust in all leave-one-out iterations, whereas the pooled FEV1 estimate was sensitive to the removal of individual studies. Therefore, the reduction in FEV1/FVC constitutes the most robust pulmonary function signal in the present analysis.

In contrast to our preliminary expectations, no statistically significant sex interaction was detected for any pulmonary function outcome (all *P* for interaction > 0.05). Although the point estimate of the FEV1/FVC reduction was larger in males (SMD = −0.31) than in females (SMD = −0.20), the between-subgroup difference was not significant (*P* for interaction = 0.468). Therefore, previously hypothesized sex-specific mechanisms—including inherent sex differences in thoracic compliance, airway caliber, and endocrine regulation (69)—were not confirmed by the present pooled data, and sex-specific claims regarding pulmonary function should be avoided until more sex-stratified evidence.

A significant regional subgroup interaction was observed for asthma (*P* for interaction < 0.001): the association in the Taiwan-based studies was substantially weaker (OR = 1.04, 95% CI: 1.01–1.06) than in studies from other regions (OR = 1.44, 95% CI: 1.29–1.62) (36, 37, 43). This disparity should be interpreted cautiously. The Taiwan studies were large registry-based studies that use claims diagnostic codes, whereas most other-region studies were smaller cross-sectional or case–control studies with partly self-reported asthma. Egger's test indicated small-study effects in the overall pool. Differences in study design, asthma ascertainment, fat distribution patterns, baseline allergic disease prevalence, and dietary habits between Eastern and Western adolescents may all contribute to this divergence (70, 71); whether it reflects true biological or environmental differences requires verification by targeted stratified research. For pulmonary function, leave-one-out analyses identified the study by Sun et al. (32) as the major source of heterogeneity for FVC, FEV1, and FEV1/FVC. This study applied a percentage-predicted scale in a very large general population sample, whereas most other studies reported absolute volumes, suggesting that differences in outcome scaling and population composition, rather than a single outlying study, drove the high heterogeneity.

No significant associations were observed for PEF, FEF75, or FEF25–75, and heterogeneity was substantial for these outcomes, with only one to four eligible studies each. Discrepancies across the existing literature mainly stem from the lack of unified diagnostic criteria and cutoff thresholds for adolescent small-airway dysfunction. The GRADE assessment rated the certainty of small-airway-related outcomes as low; hence, these results can serve only as preliminary references and should not be extrapolated to wider adolescent populations.

Several limitations in this study should be acknowledged. First, all included studies were observational, and residual confounding could not be excluded, and therefore, the pooled estimates indicated associations rather than causal effects. Second, the number of studies contributing to sex-stratified and small airway outcomes was small (one to three per subgroup), and the interaction tests had limited statistical power; the absence of significant sex interactions for pulmonary function did not exclude modest sex differences. Third, heterogeneity was very high for FVC and FEV1 (*I*^2^ > 95%), which limited clinical interpretation despite the consistent direction of effects. Fourth, asthma-related endpoints varied across studies (incident asthma, prevalent asthma, asthma control, and wheeze), contributing to indirectness. Fifth, for studies reporting medians and interquartile ranges, means and standard deviations were approximated using established methods, which may introduce additional uncertainty. Finally, funnel plot asymmetry and a significant Egger's test for the overall asthma pool suggested small study effects, although these were largely attributable to the regional subgroup structure.

Based on the included literature quality and GRADE ratings, the following recommendations are proposed. Adolescents with obesity, especially females, may be regarded as a priority population for asthma. Body mass index and pulmonary function should be monitored simultaneously in routine physical examinations. Evaluation of pulmonary function should not rely solely on FVC and FEV1, and FEV1/FVC should be included to identify early latent obstructive ventilatory dysfunction. Weight management through diet control and regular exercise should be implemented early, which may help reduce obesity-related asthma risk and progressive pulmonary function impairment. Given the moderate-to-low certainty of the overall evidence, relevant screening thresholds and intervention criteria need further verification by high-quality studies.

## 5 Strengths and limitations

### 5.1 Strengths

First, this study comprehensively evaluated the associations of overweight and obesity with asthma and multiple pulmonary function parameters among adolescents aged 10–18 years, covering large airway and mid-to-small-airway indicators (FVC, FEV1, FEV1/FVC, PEF, FEF75, and FEF25–75).

Second, sex-stratified analyses were performed using formal interaction tests rather than comparing significance within subgroups. This demonstrated a significant sex interaction for asthma but none for pulmonary function outcomes, preventing overinterpretation of sex differences.

Third, SMDs (Hedges’ *g*) were pooled instead of incomparable regression coefficients; random-effects models were prespecified; subgroup, leave-one-out, and Egger's analyses were used to explore heterogeneity and small-study effects; and the GRADE framework was applied to all outcomes, making the conclusions rigorous and transparent.

Fourth, the stratified evidence offers practical value: it can inform public health intervention frameworks for the combined burden of adolescent obesity and asthma and guide weight management, early asthma detection, and respiratory healthcare in school and primary-care settings.

### 5.2 Limitations

First, few studies contributed to sex-stratified and small-airway outcomes (one to three per subgroup). Therefore, the interaction tests had limited power, and the absence of significant sex interactions for pulmonary function does not exclude modest sex differences.

Second, all included studies were observational; residual confounding could not be excluded, and the pooled estimates indicated associations rather than causal effects. Variation in diagnostic criteria, testing procedures, and covariate adjustment produced substantial-to-very-high heterogeneity (*I*^2^ up to 98%), which was only partly explained by regional subgroup and leave-one-out analyses.

Third, asthma endpoints (incident, prevalent, control, and wheeze), pulmonary function scales (liters vs. percentage-predicted), and population composition differed across studies, contributing to indirectness; means and SDs approximated from medians and quartiles added further uncertainty.

Fourth, small-airway indicators (PEF, FEF75, and FEF25–75) were reported by a few studies with high heterogeneity, and the corresponding evidence was rated as low certainty in the GRADE assessment; these results should be interpreted cautiously.

Fifth, only sex stratification was performed; dose–response analyses by age, pubertal stage, and obesity grade were not possible, leaving the critical threshold of obesity impact unclear.

Finally, only published literature was included. Egger's test suggested small-study effects in the asthma pool—largely attributable to the regional subgroup structure, with a directionally stable trim-and-fill estimate—while tests for pulmonary function outcomes were underpowered. Therefore, publication bias cannot be fully excluded.

## 6 Conclusion

This meta-analysis indicates that overweight and obesity are associated with higher odds of asthma incidence and an early obstructive pulmonary function pattern in adolescents. The asthma association was significantly weaker in Taiwan-based registry studies than in studies from other regions (*P* for interaction < 0.001) and was significantly stronger in females (OR = 2.11, 95% CI: 1.44–3.10) than in males (OR = 1.07, 95% CI: 0.75–1.52; *P* for interaction = 0.010). The overweight/obesity group showed higher FVC and FEV1 but lower FEV1/FVC, a pattern compatible with thoracic restriction with compensatory volume preservation and subclinical airway obstruction; the reduction in FEV1/FVC was directionally consistent across all six studies and constituted the most robust finding. No statistically significant sex differences were detected for any pulmonary function outcome. This stratified evidence may support targeted respiratory screening of high-risk adolescent girls, and further multiregional studies are needed to confirm these associations and explore the underlying mechanisms.

## 7 Research prospects

Multicenter, large-sample adolescent cohort studies with unified asthma diagnostic criteria and American Thoracic Society/European Respiratory Society (ATS/ERS)–standardized spirometry reporting (using Global Lung Function Initiative [GLI] reference equations) are needed, with strict control for confounders such as age, allergy history, and exercise habits.

Original studies should report group-level means, standard deviations, and sex-stratified data so that outcomes currently supported by low-certainty evidence can be verified and their GRADE ratings upgraded. Multidimensional stratification should be used to explore dose–response relationships by obesity level and age and to define risk warning thresholds.

Mechanistic research integrating inflammatory markers, body fat distribution, and pubertal stage is recommended to elucidate the sex disparity in obesity-related asthma susceptibility, supporting strategies for obesity prevention, early asthma screening, and pulmonary protection in adolescents.

## Data Availability

The original contributions presented in the study are included in the article/Supplementary Material, and further inquiries can be directed to the corresponding author.
