# Supplementary material for: Sex differences in the association of overweight/obesity with asthma and lung function in adolescents aged 10–18 years: a meta-analysis
Source: Front Pediatr. 2026 Oct 27;14:1889125. doi: 10.3389/fped.2026.1889125 (PMC13748915; doi:10.3389/fped.2026.1889125)
Supplement: Supplementary file 1 [file Supplementaryfile1.docx]

**1.PubMed 346**

((("Adolescent"[MeSH Terms] OR adolescent[Title/Abstract] OR teenager[Title/Abstract] OR youth[Title/Abstract])) AND ("Asthma"[MeSH Terms] OR asthma[Title/Abstract] OR bronchial asthma[Title/Abstract]) AND ("Overweight"[MeSH Terms] OR "Obesity"[MeSH Terms] OR overweight[Title/Abstract] OR obese[Title/Abstract] OR obesity[Title/Abstract] OR BMI[Title/Abstract] OR body mass index[Title/Abstract]) AND ("Pulmonary Function Tests"[MeSH Terms] OR lung function[Title/Abstract] OR pulmonary function[Title/Abstract] OR FEV1[Title/Abstract] OR FVC[Title/Abstract] OR PEF[Title/Abstract]))

**2.Web of Science 875**

TS=(adolescent OR teenager OR youth) AND TS=(asthma OR bronchial asthma) AND TS=(overweight OR obese OR obesity OR "body mass index" OR BMI) AND TS=("pulmonary function tests" OR "lung function" OR "pulmonary function" OR FEV1 OR FVC OR PEF)

**3.Embase 889**

('adolescent'/exp OR adolescent OR teenager OR youth) AND ('asthma'/exp OR asthma OR 'bronchial asthma') AND ('overweight'/exp OR 'obesity'/exp OR overweight OR obese OR obesity OR 'body mass index' OR BMI) AND ('lung function'/exp OR 'pulmonary function test'/exp OR 'lung function' OR 'pulmonary function' OR FEV1 OR FVC OR PEF)

**4.Cochrane Library 45**

(adolescen* OR teenager* OR youth) AND (asthma* OR "bronchial asthma") AND (overweight* OR obes* OR "body mass index" OR BMI) AND ("lung function" OR "pulmonary function" OR "pulmonary function test" OR FEV1 OR FVC OR PEF)

**5.中国知网（CNKI）检索式 10**

主题 =(青少年 OR 青春期) AND 主题 =(哮喘 OR 支气管哮喘) AND 主题 =(超重 OR 肥胖 OR 体质指数 OR BMI) AND 主题 =(肺功能 OR 肺活量 OR FEV1 OR FVC OR 呼气峰流速)

**6.万方数据库检索式15**

(青少年 OR 青春期) AND (哮喘 OR 支气管哮喘) AND (超重 OR 肥胖 OR 体质量指数 OR BMI) AND (肺功能 OR 用力肺活量 OR 第一秒用力呼气容积 OR 呼气峰流速)

**7.维普数据库检索式14**

M=(青少年 OR 青春期) AND M=(哮喘 OR 支气管哮喘) AND M=(超重 OR 肥胖 OR 体质量指数 OR BMI) AND M=(肺功能 OR FEV1 OR FVC OR 呼气峰流速)

**8.中国生物医学文献数据库（CBM）检索式 3**

(青少年 OR 青春期) AND (哮喘 OR 支气管哮喘) AND (超重 OR 肥胖 OR 体质量指数 OR BMI) AND (肺功能 OR 用力肺活量 OR 第一秒用力呼气容积 OR 呼气峰流速)

| Quality evaluation | | | | | | | | | |
| --- | --- | --- | --- | --- | --- | --- | --- | --- | --- |
| Study | Selection(Maximum 4 stars) | | | | Comparability (Maximum 2 stars) | Outcomes(Maximum 3 stars) | | | Total |
|  | Representativeness of Exposed Cohort | Selection of Non-Exposed Cohort | Ascertainment of Exposure | Outcome Not present at the Start of the Study |  | Assessment of Outcomes | Length of  Follow-Up | Adequacy of  Follow-Up |  |
| Kong2015 | ☆ | ☆ | ☆ | ☆ | ☆☆ | ☆ | ☆ | ☆ | 9 |
| Sun2015 | ☆ | ☆ | ☆ | ☆ | ☆ | ☆ |  | ☆ | 7 |
| Wu2026 | ☆ | ☆ | ☆ | ☆ | ☆☆ | ☆ | ☆ | ☆ | 9 |
| Aris2022 |  |  | ☆ | ☆ | ☆ | ☆ | ☆ | ☆ | 6 |
| Chen2016 | ☆ |  | ☆ | ☆ | ☆☆ | ☆ | ☆ | ☆ | 8 |
| Chen2021 |  | ☆ | ☆ | ☆ | ☆☆ | ☆ | ☆ | ☆ | 8 |
| Chu2009 | ☆ | ☆ | ☆ | ☆ | ☆☆ |  | ☆ | ☆ | 8 |
| Cibella2015 | ☆ | ☆ | ☆ | ☆ | ☆ | ☆ | ☆ | ☆ | 8 |
| Ekström2018 | ☆ | ☆ | ☆ | ☆ | ☆ | ☆ | ☆ |  | 7 |
| Engwa2022 | ☆ | ☆ | ☆ |  | ☆☆ | ☆ | ☆ | ☆ | 8 |
| Ho2011 | ☆ | ☆ | ☆ | ☆ | ☆ |  | ☆ | ☆ | 7 |
| Kalm2020 | ☆ | ☆ | ☆ | ☆ | ☆☆ |  | ☆ | ☆ | 8 |
| Kang2024 | ☆ | ☆ | ☆ | ☆ | ☆ |  | ☆ | ☆ | 7 |
| Lang2019 | ☆ | ☆ | ☆ | ☆ | ☆☆ |  | ☆ | ☆ | 8 |
| Rhee 2021 |  | ☆ |  | ☆ | ☆ | ☆ | ☆ | ☆ | 6 |
| Sposato 2013 | ☆ | ☆ | ☆ | ☆ | ☆ |  | ☆ | ☆ | 7 |
| Wang2014 | ☆ | ☆ | ☆ | ☆ | ☆☆ | ☆ | ☆ | ☆ | 9 |
| Wu2024 | ☆ | ☆ | ☆ | ☆ | ☆☆ | ☆ | ☆ |  | 8 |

| GRADE quality of evidence | | | | | | | | | |
| --- | --- | --- | --- | --- | --- | --- | --- | --- | --- |
| Outcome | n | Pooled effect (95% CI) | Risk of bias | Inconsistency | Indirectness | Imprecision | Publication bias | Upgrade | **Certainty** |
| Asthma, overall | 12 | OR 1.26 (1.15, 1.37) | not seriousᵃ | not seriousᵇ | serious, −1ᶜ | not serious | suspected, −1ᵈ | none | **⊕⊕◯◯ Low** |
| Asthma, Taiwan | 3 | OR 1.04 (1.01, 1.06) | not seriousᵃ | not serious | serious, −1ᵉ | not serious | undetected | none | **⊕⊕⊕◯ Moderate** |
| Asthma, other regions | 9 | OR 1.45 (1.29, 1.62) | serious, −1ᶠ | not serious | not serious | not serious | undetectedᵍ | none | **⊕⊕⊕◯ Moderate** |
| Asthma, male | 2 | OR 1.07 (0.75, 1.52) | not seriousᵃ | not serious | serious, −1ʰ | serious, −1ⁱ | undetected | none | **⊕⊕◯◯ Low** |
| Asthma, female | 3 | OR 2.11 (1.44, 3.10) | not seriousᵃ | not serious | serious, −1ʲ | not serious | undetected | large effect, +1ᵏ | **⊕⊕⊕◯ Moderate** |
| FVC | 6 | SMD 0.35 (0.09, 0.61) | not seriousᵃ | serious, −1ˡ | serious, −1ᵐ | not serious | undetectedⁿ | none | **⊕⊕◯◯ Low** |
| FEV1 | 6 | SMD 0.23 (0.01, 0.45) | not seriousᵃ | serious, −1ˡ | serious, −1ᵐ | not seriousº | undetectedⁿ | none | **⊕⊕◯◯ Low** |
| FEV1/FVC | 6 | SMD −0.27 (−0.36, −0.18) | not seriousᵃ | serious, −1ᵖ | not serious | not serious | undetectedⁿ | none | **⊕⊕⊕◯ Moderate** |
| PEF | 3 | SMD 0.02 (−0.18, 0.22) | not seriousᵃ | serious, −1ˡ | serious, −1ᵐ | not serious۹ | undetected | none | **⊕⊕◯◯ Low** |
| FEF25-75 | 4 | SMD −0.01 (−0.12, 0.09) | not seriousᵃ | serious, −1ˡ | serious, −1ʳ | not serious۹ | undetected | none | **⊕⊕◯◯ Low** |
| FEF75 | 1 | SMD 0.02 (−0.15, 0.19) | not serious | — | serious, −1ˢ | serious, −1ᵗ | — | none | **⊕⊕◯◯ Low** |
