# Supplementary material for: Sex differences in the association of overweight/obesity with asthma and lung function in adolescents aged 10–18 years: a meta-analysis
Source: Front Pediatr. 2026 Oct 27;14:1889125. doi: 10.3389/fped.2026.1889125 (PMC13748915; doi:10.3389/fped.2026.1889125)
Supplement: Supplementary file 2 [file Supplementaryfile2.docx]

**forest plots**
